# Not All Larvae Stay Close to Home: Insights into Marine Population Connectivity with a Focus on the Brown Surgeonfish (*Acanthurus nigrofuscus*)

**DOI:** 10.1155/2011/518516

**Published:** 2011

**Authors:** Jeff A. Eble, Luiz A. Rocha, Matthew T. Craig, Brian W. Bowen

**Affiliations:** 1 Hawaii Institute of Marine Biology, University of Hawaii at Manoa, P.O. Box 1346, Kaneohe, HI 96744, USA; 2 Marine Science Institute, University of Texas at Austin, Port Aransas, TX 78373, USA; 3 Department of Marine Sciences, University of Puerto Rico Mayagüez, P.O. Box 9000, Mayagüez, PR 00681, USA

## Abstract

Recent reports of localized larval recruitment in predominately small-range fishes are countered by studies that show high genetic connectivity across large oceanic distances. This discrepancy may result from the different timescales over which genetic and demographic processes operate or rather may indicate regular long-distance dispersal in some species. Here, we contribute an analysis of mtDNA cytochrome *b* diversity in the widely distributed Brown Surgeonfish (*Acanthurus nigrofuscus*; *N* = 560), which revealed significant genetic structure only at the extremes of the range (Φ_CT_ = 0.452; *P <* .001). Collections from Hawaii to the Eastern Indian Ocean comprise one large, undifferentiated population. This pattern of limited genetic subdivision across reefs of the central Indo-Pacific has been observed in a number of large-range reef fishes. Conversely, small-range fishes are often deeply structured over the same area. These findings demonstrate population connectivity differences among species at biogeographic and evolutionary timescales, which likely translates into differences in dispersal ability at ecological and demographic timescales. While interspecific differences in population connectivity complicate the design of management strategies, the integration of multiscale connectivity patterns into marine resource planning will help ensure long-term ecosystem stability by preserving functionally diverse communities.

## 1. Introduction

The recent dramatic decline of marine ecosystems [1–3] has led to an increased interest in the use of spatially explicit management strategies, such as no-take marine reserves, to promote ecosystem stability [4–9]. Yet designing marine reserves that can support a community’s ability to absorb and recover from recurrent ecosystem disturbances requires an understanding of the scale and magnitude of population connectivity for a wide range of species and environments [9–13]. While there have been a number of recent, remarkable insights into larval dispersal distances for some taxonomic groups (e.g., Damselfishes [13, 14]), the lack of data for the majority of species continues to limit the integration of dispersal dynamics into reserve planning and design.

Most near-shore marine species exhibit an early pelagic larval phase (reviewed in [15, 16]) and larval duration has been repeatedly explored as a surrogate for species dispersal ability [17–20]. However, a comprehensive review found average pelagic larval duration (PLD) to be a poor predictor of genetic structure, and by extension dispersal ability, with previously reported correlations driven by species lacking a pelagic larval phase [21]. While a correlation between dispersal ability and general reproductive strategy may hold (i.e., brooders versus nonbrooders; reviewed in [22, 23]), there appears to be little evidence of a consistent relationship between species PLD and patterns of population connectivity [21, 24].

Recent reports emphasizing the influence of species ecology on dispersal and connectivity [25–30] may offer some insight into the discrepancy between PLD and other estimates of dispersal. For example, ecological specialists appear to be less dispersive and less successful colonizers than generalists [31]. With respect to direct larval exchange, pronounced interspecific differences in larval swimming ability and larval response to environmental signals have been identified [22, 32, 33], and inclusion of larval behavior in dispersal models can dramatically change predicted levels of local retention and larval dispersal distances [34]. The difficulty in tracking minute larvae has, however, restricted direct evaluation of dispersal distances to a small number of studies.

Whether employing induced otolith tags or multi locus parentage assignment, direct larval tracking has consistently revealed unexpectedly high levels of local larval retention [13, 14, 35–38]. In turn, this has led to the proposal that larval retention near natal sources may be a common phenomenon of reef fishes [14, 39] and of marine species in general [35]. The long-held perception that marine populations are broadly open [40–43] has now shifted towards an emphasis on the retention of larvae near source populations (reviewed in [44]), with a resulting change in recommendations for resource management [23, 37]. Yet with the exception of the Vagabond Butterflyfish (*Chaetodon vagabundus*), direct tests of larval dispersal have only been applied to fishes with small geographic ranges (<6,000 km median longitudinal range) that are restricted to either the tropical West Pacific [13, 14, 36, 37], Caribbean [35], or Mediterranean [39]. Conversely, the majority of Indo-Pacific reef fishes have longitudinal ranges exceeding 10,000 km [20], indicating that the conclusion of high larval retention may not apply to all reef species.

Dispersal ability is thought to play an important role in establishing and maintaining large geographic ranges (see [20, 45–48], but see [49, 50]). There is conflicting evidence, however, whether species’ current distributions can be used to inform spatially explicit resource planning. A comparison of dispersal distances calculated from genetic isolation-by-distance (IBD) slopes for a taxonomically diverse group of reef species [51] found no relationship between dispersal ability and species geographic range size [50]. Though, because IBD analyses assume equilibrium between migration and genetic drift (i.e., equilibrium between forces adding and culling genetic diversity), IBD-based estimates of dispersal distances have been shown to differ from known values by more than 300% [52]. Biogeographic support for a positive relationship between range size and dispersal ability can be found in the least dispersive reef species; those lacking a pelagic larval phase often have smaller geographic ranges than similar species with pelagic larval dispersal [53, 54]. Likewise, genetic assessments of Hawaiian reef fishes have consistently found endemic fishes to be genetically subdivided across the 2,600 km archipelago, while more broadly distributed species reveal a lack of barriers to gene flow over the same region (see [55–58], but see [59]).

**Figure 1:**
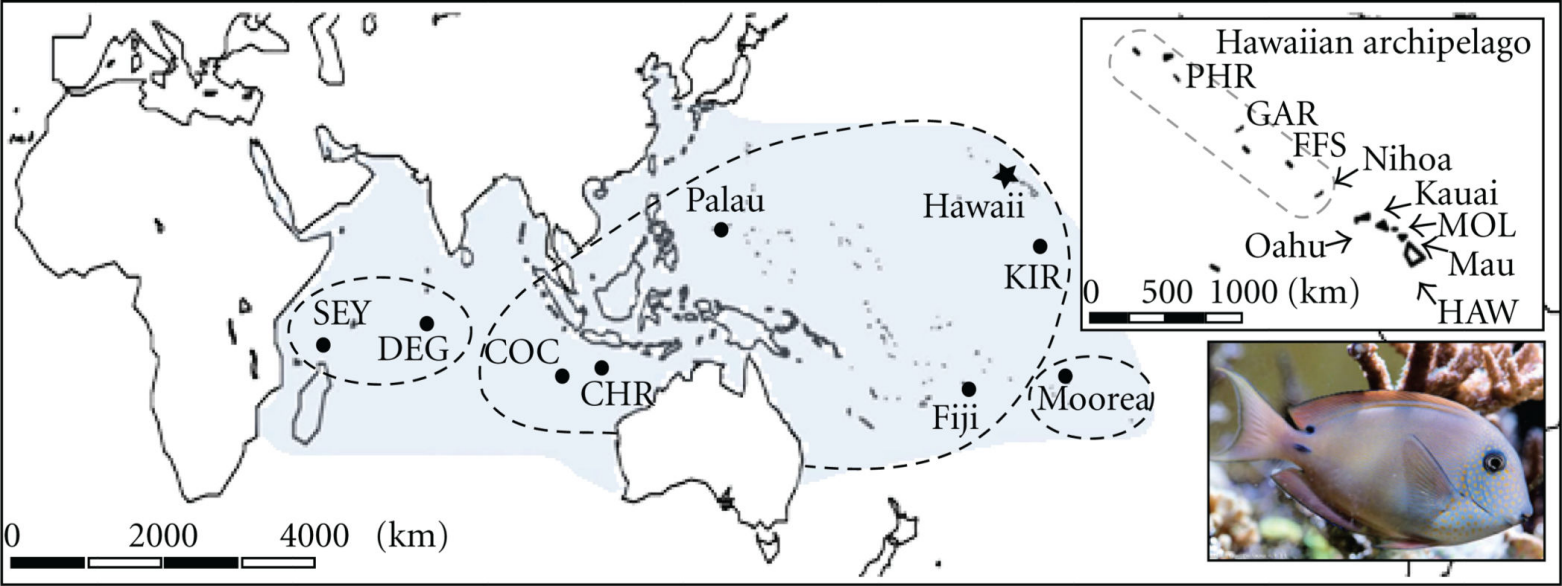
Location of Brown Surgeonfish (*Acanthurus nigrofuscus*) collection sites. Collections in Hawaii were made in June/July 2005–06, and elsewhere from September 2006 to June 2009. Inset details collections within the Hawaiian Archipelago, with the boundary of the Papahānaumokuākea Marine National Monument indicated by the dashed circle. Site abbreviations are as follows: SEY, Seychelles; DEG, Diego Garcia; COC, Cocos Islands; CHR, Christmas Island; KIR, Kiritimati; PHR, Pearl and Hermes Reef; GAR, Gardner Pinnacles; FFS, French Frigate Shoals; MOL, Molokai; HAW, Hawaii Island. Dashed borders on the main map indicate site groupings as determined in Samova. Photo credit: http://www.aquaportail.com/.

To further investigate the relationship between species range size and patterns of population connectivity, we assessed mitochondrial DNA (mtDNA) diversity in the Brown Surgeonfish (*Acanthurus nigrofuscus*) across a range that stretches from the east coast of Africa to Hawaii and Pitcairn Island in the Central Pacific [60, Figure 1]. The Brown Surgeonfish is a “generalist” herbivore that occurs in a variety of habitats from lagoons to forereefs, with feeding behavior that varies between seasons and locations [60–62]. The geographic distribution of the Brown Surgeonfish is similar to many Indo-Pacific species, covering a longitudinal distance of more than 21,000 km and straddling the well described biogeographic barrier centered on the Indo-Malay Archipelago, the Indo-Pacific Barrier (IPB [63]). A previous assessment of Brown Surgeonfish population genetic structure within Hawaii indicated extensive gene flow throughout the 2,600 km archipelago (Φ_ST_ =−0.006, *P* = .752 [64]), a pattern consistent with expectations of large-scale population connectivity in widely distributed species. In addition, we contrast phylogeographic patterns (i.e., geographic distribution of genetic diversity) from the Brown Surgeonfish and other broadly distributed fishes to those from co-occurring small range species to offer some insight into how recent findings of high larval retention can be applied to marine communities and to the development of ecosystem-based management strategies.

Similar phylogeographic comparisons have provided valuable insights into species’ life history [65, 66], ecology [67–69], and population history (see [24, 59, 65], reviewed in [70]). However, reliance on mtDNA markers presents some challenges. Of particular concern are the strikingly different temporal and spatial scales that genetic and demographic processes operate [71, 72]. Because most population genetic approaches integrate historical and contemporary processes, strong historical signals (e.g., colonization events) can obscure more recent patterns of gene flow [73, 74]. Additionally, demographic independence of populations can occur even when migration is high enough to inhibit genetic differentiation—meaning that a lack of genetic differentiation can not be taken as proof of frequent larval exchange [74, 75]. Therefore, rather than directly assessing ongoing larval exchange, we use findings from the Brown Surgeonfish to set up a qualitative assessment of the relationship between reef fish biogeography (range size) and population connectivity. While the increasing use of genomic molecular analyses continues to improve the resolution of fine-scale connectivity patterns (reviewed in [76]), we confine our comparison to mtDNA markers because their relative abundance offers opportunities for comparisons not yet possible with other markers.

## 2. Materials and Methods

Tissue collections from Hawaii (*N* = 281 [64]) were supplemented with range-wide sampling (*N* = 279; Figure 1). The combined 560 Brown Surgeonfish were collected from 17 Indo-Pacific locations with polespears while snorkeling or with SCUBA. DNA was extracted using a standard salting out protocol [77], and a 694 bp section of mtDNA cytochrome *b* was amplified using the heavy strand primer (5’-GTGACTTGAAAAACCACCGTTG-3’) from [78] and light strand primer (5’-ACAGTGCTAATGAGGCTAGTGC-3’) modified from [79]. PCR and sequencing protocols are described in [58]. In brief, polymerase chain reaction (PCR) amplification mixes consisted of 3.0 mM MgCl_2_, 0.26 µM of each primer, 50 nM dNTPs, 1.0 units *Taq* DNA polymerase and 2.0 µL of 10x PCR buffer (Bioline USA Inc., Taunton, Mass) in 20 µL total volume. PCR thermal cycling consisted of an initial denaturing step at 94° C for 1 min, followed by 35 cycles of 94, 55, and 72°C for 30 s each, with a final extension of 72°C for 10 min. Sequencing reactions with fluorescently labeled dideoxy terminators were analyzed on an ABI 3100 automated sequencer (Applied Biosystems, Foster City, Calif) in the EPSCoR genetic analysis facility at the Hawaii Institute of Marine Biology. Only rare and questionable haplotypes were sequenced in both directions. Newly derived unique haplotypes have been deposited in Gen Bank (accession numbers HQ157717–157797). Resulting sequences were aligned in Mafft 6.62 [80].

Haplotype (*h*) and nucleotide (π) diversities for each collection site were calculated in Arlequin 3.11 [81] which implements diversity index algorithms described in [82]. Differences in diversity values were assessed with a one-sided *T*-test. A statistical parsimony network of haplotypes was constructed using TCS 1.2.1 [83].

Population structure was assessed with a spatial analysis of molecular variance (Samova 1.0 see [84]). Samova removes bias in population designation by implementing a simulated annealing procedure within the analysis of molecular variance (AMOVA) framework (Arlequin 3.11) to identify maximally differentiated groupings without *a priori* assumptions of group identity. To ensure validity of the maximally differentiated groupings, the simulated annealing process was repeated 100 times from a different initial partition of samples into *K* groups. The configuration with the largest, statistically significant estimate of among group differentiations (Φ_ct_) is retained as the best sample grouping. Samova was run with values of *K* = 2 to 10 to identify the most likely number of populations. Because Samova cannot be run for *K* = 1, a separate AMOVA analysis was conducted with all collections combined into a single group. Deviations from random expectations were tested with 20,000 permutations. Patterns of pairwise genetic differentiation among individual sampling sites (Φ_st_) were estimated in Arlequin with the mutational model of Tamura and Nei [85], which was identified as the best fit to the data by the Akaike information criterion (AIC) as employed in Modeltest [86, 87]. We also calculated standardized estimates of allele frequency differences, *D*_es_t (equation 13 see [88]).

A Mantel test with 10,000 simulations was used to test for an isolation-by-distance (IBD) signature (a positive correlation between geographic and genetic distance measures [89, 90]). To provide insight into how the spatial scale of gene flow may differ across potentially interconnected islands and across large stretches of open ocean, IBD tests were conducted separately on the full data set and within Samova-defined populations.

We tested for a signature of population expansion with Fu’s *F_s_* [91] and by comparing observed and expected pairwise mismatch distributions [92] in Arlequin with 90,000 simulated samples. Fu [91] noted that *F_s_* is particularly sensitive to deviations from a constant population size, with population expansion resulting in a significant, negative *F_s_*. If there was evidence of population expansion, we estimated ancestral and contemporary female effective population sizes (*N*_ef_) from the equation: θ = *N*_ef_2 μ*t*, with μ being the estimated annual fragment mutation rate and *t* is the estimated generation time. Estimates of pre- and post expansion θ were derived from the sudden expansion model of the mismatch distributions. Population ages in years were estimated from the population age parameter (τ), with τ = 2 μ*T*, where *T* is the time since the most recent bottleneck. We provisionally applied a generation time of 5 years for the Brown Surgeonfish [92] and a within lineage, annual-per-site mutation rate of 1.55 × 10^−8^ per year [93].

## 3. Results

Cytochrome *b* sequencing revealed 110 closely related haplotypes (*h* = 0.65–0.91; π = 0.0017–0.0045 Table 1). Haplotype diversity in Hawaii (*h* = 0.65–0.87) is similar to other peripheral collections (Seychelles and Moorea, *h* = 0.72–0.78) but is significantly lower than central Indo-Pacific collections (Diego Garcia to Kiritimati, *h* = 0.85–0.89; one way *T*-test, *P <* .001).

**Figure 2:**
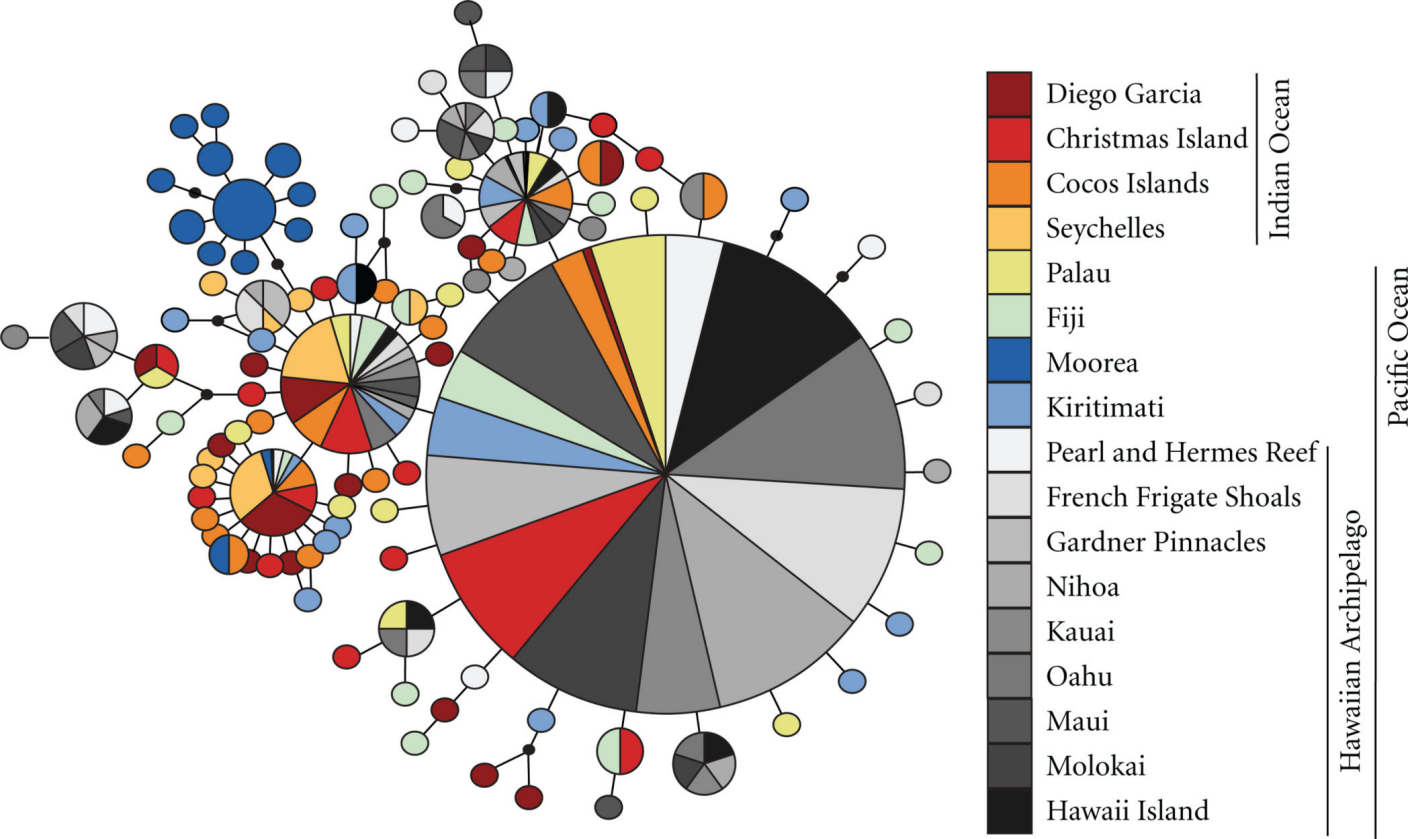
Statistical parsimony network for the Brown Surgeonfish. Area of circles is proportional to the frequency of the respective haplotype. Black dots represent missing haplotypes and colors represent haplotype location (see key). (1,200 km northeast of Moorea) where it is either very rare or absent (M. Gaither pers. comm.). Gaither et al. [68] argued that population and community level differences between Moorea (Society Islands) and the Marquesas resulted from limited dispersal across the fast flowing Southern Equatorial Current (SEC), a proposal consistent with recent evidence indicating that ocean currents can have a strong influence on population connectivity [99, 100]. Considering that the Society Islands are located below the westward flowing SEC and within the southerly flowing South Pacific Current [101], oceanographic isolation may likewise explain the concordant break between Moorea and other central Pacific sampling sites observed in both the Bluestriped Snapper and Brown Surgeonfish.

The statistical parsimony network demonstrates both the high diversity and low differentiation of haplotypes collected from sites distributed across two ocean basins (Figure 2). Abundant haplotypes are well dispersed through most sites with the exception of Moorea in French Polynesia, which exhibited a high number of geographically restricted haplotypes.

Samova identified three maximally differentiated groupings, with significant population differentiation occurring only at eastern and western edges of the range (Φ_ct_ = 0.452, *P <* .001; Table 2). Central Indo-Pacific collections (Cocos Islands to Hawaii) form one large group, while Moorea is isolated in the South Pacific and Diego Garcia is grouped with the Seychelles in the Indian Ocean (Figure 1). Estimates among group differentiations (Φ_ct_) dropped incrementally with the addition of *K >* 3 populations (data not shown) indicating a lack of further population subdivision. Pairwise Φ_st_ and *D*_est_ highlight both the isolation of Moorea as well as a pattern of increasing divergence in the Indian Ocean with distance from the IPB (Table 3). Both the Seychelles and Diego Garcia were significantly different from all other locations, though the test for IBD within this region was nonsignificant (*R^2^* = 0.46, *P* = .16). Pairwise estimates of Φ_st_ within the Samova-defined central Indo-Pacific population were near zero. Nonetheless, there was a clear IBD signature across this broad region (*R^2^* = 0.61, *P <* .001, slope = 2.0 × 10^−5^, y-intercept = 0.038) even though Christmas Island, located in the western Indian Ocean, was only marginally different from Hawaiian collections (Φ_st_ = 0.007–0.032, *P* = .030–.246; *D*_est_ =−0.04–0.166, *P* = .016–.618) and was genetically indistinguishable from Kiritibati, Fiji, and Palau.

Tests for demographic expansion were run on the three Samova populations. While a significant deviation between simulated and observed mismatch distributions was observed only in the Moorean collection (SSD = 0.173, *P* = .01), Fu’s *F_s_* was significantly negative in all three populations (Table 4). Simulations have shown *F_s_* to be more sensitive to recent population expansion than other tests of demographic history [91], so we place greater emphasis on these tests as indicators of population expansion. Mismatch analyses indicate the time since last expansion to be on the order of 56,000 and 24,000 years in Moorea and the Seychelles/Diego Garcia, respectively, and 83,000 years in the central Indo-Pacific (Table 4). Estimates of post-expansion female effective population size derived from θ_1_ were unresolved in both the Seychelles and Moorea, but was approximately 300,000 in the central Indo-Pacific population (Table 4).

## 4. Discussion

Several patterns are apparent from the phylogeographic assessment of the Brown Surgeonfish. First, populations are characterized by clusters of closely related haplotypes, high haplotype diversity and low nucleotide diversity, a common pattern in reef fishes (Figure 2, Table 1; [94]). Second, Samova and pairwise Φ_st_ and *D*_est_ indicate that distances as long as 11,000 km do not appear to be much of an obstacle to gene flow in the Brown Surgeonfish (Table 3). Kiribati and Fiji are located at 11,900 and 8,800 km, respectively from Cocos Islands in the Eastern Indian Ocean, yet there were no indications of population differentiation across this large expanse of interspersed islands and reefs. Third, Fu’s *F_s_* and coalescence estimates indicate population contractions throughout the Brown Surgeonfish’s range during the most recent glacial period (∼110–10 ka [95]), with subsequent population expansion, and in turn, increasing genetic diversity (Table 4).

Genetic evidence of postglacial population expansion is common in reef fishes and indicates broad ecosystem level responses to global climate change [94]. These patterns also demonstrate the temporal durability of historical genetic signals and the difficulty extrapolating demographically meaningful estimates of connectivity from genetic data. In particular, population expansion can prolong the time required for populations to reach equilibrium between the forces adding and culling genetic diversity, potentially resulting in an overestimate of population connectivity [96]. There is, however, a negative correlation between rates of gene flow and the time required for populations to attain equilibrium, meaning that well-connected populations will reach equilibrium, and therefore return accurate connectivity estimates, more rapidly than similar populations that are less well connected [90, 97]. Accordingly, while evidence of population expansion in the Brown Surgeonfish may indicate that population connectivity across the central Indo-Pacific may be overestimated, the presence of highly differentiated populations at the edge of the species range (Table 2) demonstrates ample opportunity for the establishment of genetic differentiation within the central Indo-Pacific should gene flow be truly restricted across this region. Likewise, the presence of an IBD signature within the central Indo-Pacific argues against the overestimation of gene flow, as IBD will arise only as populations approach equilibrium [98]. We therefore conclude that the lack of genetic differentiation observed across the majority of the Brown Surgeonfish’s range is indicative of high population connectivity rather than a temporal artifact of nonequilibrium conditions.

The population structure of the Brown Surgeonfish is remarkably similar to the widely distributed Bluestriped Snapper (*Lutjanus kasmira*), which differs only in having an additional genetic break between Moorea and the Marquesas [68]. Notably, the Brown Surgeonfish is abundant throughout French Polynesia with the exception of the Marquesas

The lack of pronounced mtDNA genetic structure across the central Indo-Pacific in both the Brown Surgeonfish and Bluestriped Snapper has been observed in a growing number of widely distributed reef fishes. Of the six published genetic surveys of reef fishes with geographic ranges extending from Africa to the East Pacific (27,000 km), all but the Convict Surgeonfish (*Acanthurus triostegus*) revealed little to no genetic subdivision within the Central and West Pacific [56, 68, 102–105]. Remarkably, there was no evidence of population subdivision in the Blue-spine Surgeonfish (*Naso unicornis*) from the West Indian Ocean to French Polynesia in the South Pacific [104], in the trumpetfish, (*Aulostomus chinensis*,) from West Australia to Panama [102], and in the Yellow-edged Moray (*Gymnothorax flavimarginatus*) from East Africa to the East Pacific [105].

The broad genetic connectivity consistently observed in the most widely distributed Indo-Pacific fishes highlights an emerging relationship between reef fish range size and genetic connectivity. In particular, Soldierfishes (genus *Myripristis* [24, 56]), Pygmy Angelfishes (genus *Centropyge* [106, 107]), Trumpetfishes (genus *Aulostomus* [102]), Unicornfishes (genus *Naso* [104, 108]), Moray Eels (genus *Gymnothorax* [105]), and at least some Snappers (genus *Lutjanus* [68]) and Surgeonfishes (genus *Acanthurus* [31, 109]) have demonstrated an ability to maintain genetic homogeneity across tens of thousands of kilometers.

For widely distributed species, genetic homogeneity across much of the Indo-Pacific is largely concordant with biogeographic provinces and barriers, previously defined based on species distributions. For example, the region stretching from the eastern Indian Ocean to the Central Pacific is recognized by biogeographers as the Indo-Polynesian province [110–112]. Hobbes et al. [113] observed that many Indian and Pacific reef fishes overlap at our sample location in the Eastern Indian Ocean (Christmas Island), and in some cases hybridize there. For large-range fishes, this location is commonly the western limit of a broad central Indo-Pacific population, and hence demonstrates the alignment of intraspecific phylogeographic patterns with biogeographic provinces.

This pattern of widely distributed reef fishes successfully bridging long-distances contrasts starkly with the remarkably complex patterns of population structure commonly observed in smaller range fishes. The leopard coral grouper (*Plectropomus leopardus*) is restricted to reefs from West Australia to Fiji, and a survey of mtDNA control region diversity indicated deep genetic partitions, with a minimum of six highly differentiated populations (F_st_ = 0.90 to 0.94 [114]). Likewise, a comparative mtDNA survey of three restricted-range Damselfishes (*Amphiprion melanopus, Chrysiptera talboti* and *Pomacentrus moluccensis*) and the restricted-range Wrasse, (*Cirrhilabrus punctatus,*) revealed concordant morphological and genetic differentiation, and indicates evolutionary level partitions among the closely linked reefs of the West Pacific [115]. Small range species with planktonic larval dispersal and pronounced mtDNA population subdivision appear to be particularly common in Damselfishes (genus *Amphiprion* [115–117]; genus *Chrysiptera* [115, 118]; genus *Dascyllus* [57, 119]; genus *Pomacentrus* [119]), and Groupers (genus *Epinephelus* [55, 114]; genus *Plectropomus* [114]).

For species occurring within the central Indo-Pacific, broad genetic homogeneity should be facilitated by stepping stone gene flow across the regions relatively abundant reefs. Indeed, dispersal can be accomplished across this vast region without having to traverse more than 800 km of open ocean [120], yet even these relatively limited distances act as effective barriers to gene flow in many smaller range fishes (e.g., [114]). Conversely, the limited genetic subdivision commonly observed in widely distributed species indicate larval exchange across thousands of kilometers of open ocean, including across the world’s largest marine biogeographic barrier, the Eastern Pacific Barrier (EPB [63, 121, 122]. The EPB comprises the 4,000 to 7,000 km expanse of open ocean separating the islands of the Central Pacific from the west coast of the Americas. The suite of species demonstrating recent or ongoing larval connectivity across the EPB contains members of the most common Indo-Pacific reef fish families, though notably absent are Damselfishes (Pomacentridae) and Groupers (Serranidae), identified above as taxanomic families with limited individual and geographic range.

It has been noted that mtDNA markers may not accurately reflect population histories, due to either direct selection on the marker or adjacent DNA segments [123]. However, when comparing genetic partitions among multiple species, matching patterns indicates such concerns are likely unwarranted since the biological or environmental mechanisms that drive concordance across species will likewise drive concordance across markers [70]. A notable exception to this rule is sex-biased dispersal [124, 125], though this is unlikely to be an issue in species with pelagic larval dispersal [68]. Accordingly, the broad agreement between biogeographic and phylogeographic patterns herein demonstrates that species differ in dispersal ability at biogeographic and evolutionary time-scales, which in turn would appear to indicate that species differ in the extent and magnitude of population connectivity at demographic and ecological time-scales.

How do these findings translate into policies for ecosystem-level management of reef communities? The debate continues over the extent to which genetic connectivity relates to demographic connectivity [126]. Further application of larval tracking will help identify the degree to which species and regions may differ in the scale and extent of larval exchange. However, until that time, the apparent relationship between reef fish range size and extent of genetic connectivity indicates that recent evidence of high local larval retention may only apply to species with small geographic ranges. In all likelihood, marine communities contain species with markedly different dispersal abilities [127–129], including many capable of larval exchange over thousands of kilometers.

While interspecific differences in realized dispersal would appear to complicate the development of effective marine reserves, reserve design may be simplified by focusing on the species that show the finest scale of genetic isolation [69, 130, 131]. Emphasis on short-distance dispersers requires protecting reefs on a local scale, resulting in the establishment of a network of smaller reserves [129]. While setting aside one or a few larger reserves might be politically more expedient than placing an equivalent area under protection with multiple small reserves [132], a network of smaller reserves would better accommodate differences in dispersal ability among resident species by facilitating linkages among reserves at multiple spatial scales. Under this scenario, protected populations would be sustained by either local retention within the reserve or dispersal between adjacent reserves for short to moderate dispersing species, and by larval exchange between distant reserves for widely dispersing species. Additionally, a network of smaller reserves would maximize opportunities for larval subsidy to unprotected populations by increasing the likelihood of seeding reefs outside of reserve boundaries [133], one of the primary goals of reserves designed to mitigate fisheries impacts [134]. No matter the management strategy employed, overcoming the challenges of incorporating multiscale connectivity patterns into resource management planning will ultimately help ensure long-term resource stability by preserving communities of species that differ markedly in how they respond to local and global environmental impacts.

## Figures and Tables

**Table 1:** Brown Surgeonfish collection sites with sample size, haplotype diversity (*h*), and nucleotide diversity (π) per sample and for the overall data set (Total), with standard deviations in parentheses.

Location	*N*	*h*	π
Pearl and Hermes Reef	20	0.87 (0.06)	0.0045 (0.0027)
Gardner Pinnacles	25	0.72 (0.08)	0.0021 (0.0014)
French Frigate Shoals	33	0.72 (0.08)	0.0021 (0.0013)
Nihoa	40	0.73 (0.06)	0.0026 (0.0016)
Kauai	26	0.81 (0.06)	0.0023 (0.0015)
Oahu	39	0.70 (0.07)	0.0019 (0.0013)
Molokai	29	0.71 (0.08)	0.0024 (0.0016)
Maui	36	0.77 (0.06)	0.0030 (0.0018)
Hawaii Island	33	0.65 (0.09)	0.0022 (0.0015)
Kiritimati	35	0.89 (0.04)	0.0032 (0.0020)
Fiji	30	0.91 (0.03)	0.0031 (0.0020)
Palau	36	0.90 (0.03)	0.0028 (0.0018)
Moorea	31	0.78 (0.08)	0.0030 (0.0019)
Christmas Island	51	0.85 (0.03)	0.0025 (0.0016)
Cocos Islands	32	0.88 (0.04)	0.0030 (0.0019)
Diego Garcia	32	0.85 (0.05)	0.0030 (0.0017)
Seychelles	32	0.72 (0.07)	0.0017 (0.0012)

Total	560	0.85 (0.01)	0.0032 (0.0020)

**Table 2:** Structural analysis of molecular variance (Samova) with maximally differentiated groupings for (*K* = 1 to 3) and percent variation (%Var.) and fixation indices (Φ).

Number of groups	Groupings	Among groups		Among samples within groups		Among samples
		Φ_CT_	%Var.	Φ_SC_	%Var.	ΦST
1	All sites			0.201‡	20.06	
2	Moorea/All other sites	0.563	56.28	0.082‡	3.59	0.599‡
3	Moorea/Seychelles, Diego Garcia/All other sites	0.452‡	45.24	0.053‡	2.91	0.481‡

Significance represented by:

‡ *P*≤ .001.

**Table 3:** Results of pairwise tests for population structure with Φ_st_ (below diagonal) and D_est_ (above diagonal).

	PHR	FFS	GAR	Nihoa	Kauai	Oahu	MOL	Maui	HAW	KIR	Fiji	Palau	Moorea	CHR	COC	DEG	SEY
PHR	∼	0.031	0.059	0.032	−0.005	0.015	0.015	−0.020	0.059	0.225	0.098	0.028	**0.982**‡	0.021	0.319*	0.611‡	**0.695**‡
FFS	0.033	∼	−0.031	−0.004	0.008	−0.030	−0.040	−0.036	−0.005	0.341‡	0.267†	0.187*	**0.988**‡	0.134*	**0.461**‡	**0.805**‡	**0.828**‡
GAR	0.020	−0.025	∼	−0.053	−0.034	−0.021	−0.044	−0.020	0.007	0.151	0.135	0.114	**0.967**‡	0.062	0.283	**0.818**‡	**0.816**‡
Nihoa	0.007	−0.001	−0.013	∼	−0.022	−0.026	−0.045	−0.023	−0.023	0.200*	0.184*	0.147*	**0.971**‡	0.099	0.348†	**0.862**‡	**0.887**‡
Kauai	0.022	−0.015	−0.022	−0.017	∼	−0.029	−0.027	−0.052	0.046	0.062	−0.001	0.008	**0.970**‡	−0.041	0.148	**0.674**‡	**0.675**‡
Oahu	0.027	−0.007	−0.010	−0.008	−0.024	∼	−0.049	−0.034	−0.027	0.258†	0.195*	0.139*	**0.981**‡	0.079	0.362‡	**0.763**‡	**0.775**‡
MOL	0.017	−0.009	−0.013	−0.018	−0.026	−0.018	∼	−0.053	−0.027	0.260*	0.218*	0.165*	**0.978**‡	0.106	0.397†	**0.837**‡	**0.868**‡
Maui	0.008	−0.007	−0.013	−0.015	−0.019	−0.008	−0.021	∼	0.019	0.234*	0.168*	0.130	**0.980**‡	0.072	0.345†	**0.762**‡	**0.781**‡
HAW	0.006	0.000	−0.002	−0.015	−0.005	−0.003	−0.010	0.002	∼	0.333†	0.296†	0.219†	**0.983**‡	0.165*	**0.473**‡	**0.859**‡	**0.895**‡
KIR	0.037	0.016	−0.001	0.020	0.001	0.017	0.019	0.016	0.027	∼	−0.124	−0.014	**0.943**‡	0.012	−0.068	**0.623**‡	**0.628**‡
Fiji	0.016	0.004	−0.010	0.007	−0.005	0.004	0.006	0.004	0.010	−0.012	∼	−0.085	**0.953**‡	−0.078	−0.097	0.477‡	**0.496**‡
Palau	0.027	0.025	0.017	0.040*	0.026	0.040*	0.041*	0.040*	0.029	0.003	−0.007	∼	**0.947**‡	−0.058	0.002	0.381†	**0.486**‡
Moorea	**0.497**‡	**0.615**‡	**0.611**‡	**0.599**‡	**0.608**‡	**0.644**‡	**0.609**‡	**0.582**‡	**0.608**‡	**0.563**‡	**0.554**‡	**0.566**‡	∼	**0.960**‡	**0.930**‡	**0.952**‡	**0.960**‡
CHR	0.029	0.018	0.007	0.031*	0.015	0.030*	0.033*	0.031*	0.022	−0.002	−0.010	−0.012	**0.583**‡	∼	0.024	0.444‡	**0.472**‡
COC	0.051*	0.091‡	0.073*	0.100‡	0.085‡	0.117‡	0.110‡	0.092‡	0.101‡	0.023	0.022	0.006	**0.536**‡	0.012	∼	0.304*	0.318†
DEG	**0.174**‡	**0.288**‡	**0.281**‡	**0.295**‡	**0.292**‡	**0.335**‡	**0.310**‡	**0.278**‡	**0.293**‡	**0.187**‡	**0.189**‡	**0.146**‡	**0.538**‡	**0.172**‡	0.065†	∼	0.022
SEY	**0.240**‡	**0.355**‡	**0.357**‡	**0.359**‡	**0.369**‡	**0.407**‡	**0.385**‡	**0.339**‡	**0.366**‡	**0.236**‡	**0.244**‡	**0.196**‡	**0.583**‡	**0.218**‡	0.101†	−0.010	∼

Significance represented by:

* for *P* < .05,

† for *P* < .01,

‡ for *P* < .001, and bold for *P* ≤ .0004 (Bonferroni correction for multiple comparisons). Location abbreviations as described in Figure 1.

**Table 4:** Estimates of historical demography including pre- and post-expansion theta (*θ*_0_ and *θ*_1_), female effective population size (*N*_ef_), mismatch analyses (sum of squared deviations; SSD), tau (τ), time since last bottleneck (age in years), and test for population expansion (*F_s_*), with unresolved values shown as NR. Standard deviations for population parameter estimates are given in parentheses.

	Mismatch distribution							
SAMOVA populations	θ_0_	*N*_ef0_	θ_1_	*N*_ef1_	SSD	*t*	Age	*F_s_*
Central Indo-Pacific	0.014 (0–0.745)	130 (0–7,000)	30.57 (4.47–NR)	284,000 (42,000–NR)	0.003	1.78 (0.82–2.52)	83,000 (38,000–117,000)	−26.73‡
Moorea	0.000 (0–0.105)	0 (0–1,000)	NR (6.02–NR)	NR (56,000–NR)	0.173*	1.20 (0.37–2.05)	56,000 (17,000–95,000)	−9.38‡
Seychelles/Diego Garcia	0.009 (0–0.345)	100 (0–3,000)	NR (2.60–NR)	NR (24,000–NR)	0.002	0.52 (0.02–1.12)	24,000 (1,000–52,000)	−5.74‡

Significance represented by:

* *P*≤ .05,

‡ *P*≤ .001.
